# SUMO mediated regulation of transcription factors as a mechanism for transducing environmental cues into cellular signaling in plants

**DOI:** 10.1007/s00018-020-03723-4

**Published:** 2021-01-16

**Authors:** Dipan Roy, Ari Sadanandom

**Affiliations:** Department of Biosciences, Durham University, Stockton Road, Durham, DH1 3LE UK

**Keywords:** SUMO, Transcription factors, Cell signalling, Post-translational modifications, Gene expression

## Abstract

Across all species, transcription factors (TFs) are the most frequent targets of SUMOylation. The effect of SUMO conjugation on the functions of transcription factors has been extensively studied in animal systems, with over 200 transcription factors being documented to be modulated by SUMOylation. This has resulted in the establishment of a number of paradigms that seek to explain the mechanisms by which SUMO regulates transcription factor functions. For instance, SUMO has been shown to modulate TF DNA binding activity; regulate both localization as well as the abundance of TFs and also influence the association of TFs with chromatin. With transcription factors being implicated as master regulators of the cellular signalling pathways that maintain phenotypic plasticity in all organisms, in this review, we will discuss how SUMO mediated regulation of transcription factor activity facilitates molecular pathways to mount an appropriate and coherent biological response to environmental cues.

## Introduction

Transduction of environmental cues into cellular signalling is critical for the survivability and development of adaptive responses in any organism. An individual organism can be simultaneously subjected to multiple environmental and physiological cues and a dynamic network of interconnected signal transduction cascades decode and integrate these signals into a coherent response. Due to their sessile nature, plants heavily depend on an intricate network of signalling pathways that allows them to perceive the changes in their environment and mount an appropriate adaptive response to maintain phenotypic plasticity [1, 2]. Transcription factors (TFs) collectively constitute as master regulators of these signalling pathways that control the response of plants towards both external stimuli and internal cues [3–5]. Activation of a signalling pathway is often characterized by the binding of a ligand (which may be exogenous or endogenous in nature) to a transmembrane receptor which in turn relays the signal to cytoplasmic transducers resulting in amplification of the signal, with the endpoint of the pathway more often than not being either activation or inhibition of the activity of transcription factors [6]. Thus, essentially, transcription factors link the event of exogenous signal perception to the development of the effector response towards the signal by modulation of the expression of downstream effector genes. Moreover, the activity of dedicated transcription factors implicated specifically for a signalling pathway, allows the concerned signal transduction pathway to discriminate amongst a wide panoply of signals. Therefore, control of the expression and activity of transcription factors at the transcriptional, post-transcriptional and post-translational level due to the position of TFs in signalling networks have been proposed to underlie a master regulatory switch by which the cellular response towards environmental cues can be controlled and modulated simultaneously by several signalling pathways[7]. Post-translational modification of proteins involved in a cellular signalling pathway allows for the rapid reprogramming of the activity of the proteins and thereby provides a conduit for the environment to influence the signalling pathway. In this regard, post-translational modification by SUMO (small ubiquitin-like modifier) conjugation is emerging fast as a dynamic mechanism by which the activity and functions of transcription factors can be regulated in a cell. Indeed, numerous substrates for SUMO modifications have been linked to proteins involved in the regulation of transcription/chromatin dynamics [8]and transcription factors[9]. Recent Gene Ontology (GO) analysis of a human SUMO proteome study indicates that over 50% of sequence-specific DNA binding transcription factors implicated as activators or repressors of transcription by RNA polymerase II are conjugated to SUMO [10]. SUMO conjugation influences transcription factor functions through multiple ways that include: (a) influencing the DNA binding activity, (b) promotion of clearance of the TF from chromatin, (c) alteration of the extent of association of the transcription factor to its associated target binding sites on chromatin, (d) regulating the abundance and/or localization of the TF, (e) altering the capacity of the transcription factor to interact with transcriptional regulators such as co-repressors or co-activators and, (f) through interplay with other post- translational modifications that can influence TF activity [11].

Irrespective of whether SUMO conjugation directly or indirectly impacts the function of a TF in the context of its ability to bind DNA, the process of SUMO conjugation to transcription factors profoundly impacts the expression of downstream target genes and alters the fate of a signalling pathway. This review aims to discuss the implications of SUMO conjugation as a mechanism to modify and regulate the transcriptional output of cellular signalling pathways in response to environmental cues, with emphasis on the SUMOylation of transcription factors involved in the various cellular signalling pathways. We will first set the scene by having a brief overview of SUMO and its isoforms and the key players of the SUMO conjugation/ deconjugation machinery in plants. We will also assess how the pool of SUMO proteins and the turnover of SUMOylated proteins are maintained in a cyclical manner (SUMO cycle) in plants. We will then finally examine the well- documented examples of SUMOylation mediated modulation of the functions of transcription factors and its outcome on various cellular signalling processes in plants and also assess whether these examples in plants re-enforce the established paradigms of SUMOylation mediated modulation of gene expression and cell signalling.

## Key components of the SUMO conjugation and deconjugation machinery in plants

### Small ubiquitin-like modifier (SUMO)

SUMO proteins are ubiquitously found in all eukaryotes including single-celled eukaryotes *Saccharomyces* cerevisiae, mammals and plants. The indispensability of SUMO proteins for eukaryotic cell viability and survival is evident from the fact that deletion of the only SUMO isoform, SMT3, in yeast cells results in the loss of cell viability[12, 13] and *Arabidopsis thaliana sumo1sumo2* deletion mutants are embryonic lethal[14, 15]. SUMO protein is an 11 kDa protein and structurally similar to ubiquitin containing a signature fold called the β-grasp fold which has a β sheet with 5 anti-parallel β strands and a single helical element connecting strand β-4 and β-5 [16, 17]. The conjugation of SUMO at lysine residues of its target proteins occurs in a manner similar to ubiquitination. Similar to the ubiquitin system which regulates the majority of the cellular processes in which it is involved through diverse mechanisms involving proteolysis and signal transduction mechanisms that modulate protein–protein interactions and target protein functions, the functional implications of SUMOylation is also highly diverse. SUMO conjugation to substrate proteins can: (a) protect the protein from degradation by occluding the lysine residues that can be ubiquitinated, (b) change the localization of the substrate protein and (c) alter the interaction of the substrate protein with its cognate partner which can be other proteins or nucleic acids[18]. However, SUMO conjugation to a cognate substrate protein can also lead to the degradation of the protein if poly SUMO chains consisting of multiple SUMO moieties are appended to one single lysine residue of the target protein[19]. Evidently, proteins with poly SUMO chains are targeted by a novel class of ubiquitin E3 ligases known as STUbLs (SUMO-targeted ubiquitin Ligases), which can ubiquitinate these proteins and target them for degradation [20–24]. Thus, a combination of multiple factors including the identity of the substrate proteins that are being SUMOylated, the position of the lysine residues of the substrate proteins that are being SUMOylated and the number of SUMO substrates that are being appended to the target protein including whether multiple SUMO monomers are attached to multiple lysine residues of a target protein or poly SUMO chains are attached to a single lysine residue of a target protein, contribute to the functional outcome of SUMOylation.

#### The SUMO isoforms

The existence of multiple distinct isoforms of SUMO confers diversity to the process of SUMOylation of target proteins and also contributes to the dynamicity of molecular consequences of SUMOylation. Modulation of the function of a target protein by a specific isoform of SUMO may act as a critical regulatory point in a molecular signalling pathway where identification of the SUMO isoform conjugated to the target protein may act as a “specificity checkpoint” that allows or disallows the downstream steps of the molecular pathway to proceed. Eight SUMO isoforms are encoded by the Arabidopsis genome and amongst them, only the SUMO1, 2, 3 and 5 isoforms are expressed [25–27]. SUMO1 and SUMO2 isoforms are closely related and share 83% amino acid sequence identity, whereas the SUMO3 and SUMO5 isoforms display only 42 and 30% amino acid sequence identity with SUMO1, respectively[28]**.** This diversification between the SUMO isoforms may have implications on the dynamics of SUMOylation in plants as it involves residues that perform key molecular functions involving interactions with the key enzymes of the SUMO conjugation system, E1 and E2 and as well as interactions with the SUMO-interacting motif (SIM). SIMs are characterized by hydrophobic residues flanked by acidic residues or residues that can be phosphorylated [29, 30]. SUMO1/2 isoforms are indispensable in Arabidopsis[14] and are involved in multiple molecular pathways that ensure plant phenotypic plasticity and viability, while the biological function of SUMO3 isoform seems to be restricted to plant immune responses. As discussed in detail later in this review, one of the key roles of SUMO3 in plant defense involves interaction and modification of the protein NPR1 (NON EXPRESSOR OF PATHOGENESIS RELATED GENES1), which in turn is a master regulator of basal and systemic acquired resistance[31]. However, *sumo3* knock out plants are not impaired in resistance to infection by *Pseudomonas syringae* pv tomato DC3000 (PstDC3000) [15] suggesting that other isoforms of SUMO may be able to substitute for SUMO3 functions. The biological function of SUMO5 is yet to be elucidated although SUMO5 is evolutionarily conserved in plants [27]. On the other hand, while genomic analysis using animal and yeast counterparts of the SUMO conjugation system as queries has led to the identification of four other SUMO coding genes, SUMO 4,6, 7 and 8; Reverse transcription (RT)-PCR-based analysis indicate that SUMO 4, 6 and 7 loci are not expressed and also lack the C-terminal di-Glycine motif [7, 13], implying that the SUMO1/2/3 isoforms are the key players in plants from a functional standpoint.

#### The E1 activating enzyme

The E1 SUMO activating enzyme is a heterodimer consisting of a large subunit SAE2 (SUMO activating Enzyme2) and a small subunit SAE1 (SUMO activating Enzyme1). There are two isoforms of SAE1, SAE1a or SAE1b and either of the two isoforms can be involved in the formation of the heterodimer with SAE2. SAE2 in turn consists of four functional domains: adenylation, catalytic cysteine, UFD (ubiquitin-fold domain) and C-terminal domains[32]. The adenylation domain is the most conserved domain and the only domain the functioning of which depends on heterodimer formation. The cysteine and UFD domains on the other hand are more divergent[33]. Knockout mutations of SAE2 are embryonic lethal[14]**.** On the other hand**,** although T-DNA mutants of the SAE1a isoform are viable [14], kinetic analysis of reconstituted SUMO conjugation assays indicates that the rates of SUMO conjugation by the SAE2/SAE1a holoenzyme are higher than that of the SAE2/SAE1b in vitro [33]. Moreover, SAE1a and SAE1b exhibit different subcellular distributions and the ability to accumulate SUMO conjugates upon heat and drought stress is impeded in Arabidopsis plants lacking SAE1a [33]. These findings from this study indicate that SUMO conjugation can be influenced in vivo by E1 through a mechanism putatively occurring downstream of SUMO activation but intriguingly dependent on the identity of the SAE1 isoform that associates with SAE2 to form the E1 holoenzyme.

#### The E2 conjugating enzyme

The SUMO E2 conjugating enzyme is involved in the transfer of activated SUMO to a consensus acceptor lysine residue of the target substrate protein. Like ubiquitin-conjugating enzyme, SUMO E2 conjugating enzymes also display a characteristic fold consisting of four α-helices and four β-strands containing a signature histidine-proline-asparagine (HPN) tripeptide motif followed by the catalytic cysteine residue at the 7th or 8th position and a tryptophan residue at the 16th position (up to 29th position) from the C-terminal end of the HPN motif[34]. Like in humans and yeast, a single gene encodes for SUMO E2 conjugating enzyme in Arabidopsis referred to as SUMO conjugating enzyme 1 (SCE1). Null T-DNA insertion mutants in *SCE1* and *SAE2* (*sce1* and *sae2*) are embryo lethal in Arabidopsis with the embryos being arrested in early embryonic stages (globular, heart, early torpedo) and this indicates the indispensability of both E1 and E2 for SUMO modifications of target proteins[14]. SCE1 shares 63% identity with its human ortholog (HsUBC9) and shows 58% sequence similarity with its yeast ortholog (ScUBC9) and is one of the most conserved members of the SUMO conjugation machinery. Indeed, AtSCE1 can conjugate human SUMO1 to mouse RanGAP in vitro and substitution of the catalytic domain cysteine to serine suppresses the SUMO conjugation activity of AtSCE1[35]. The mRNA transcripts of SCE1 along with SAE1a/b are detectable in all Arabidopsis tissues including shoot tip, root, cotyledons, seedlings, the stem, siliques and flower and AtSCE1 colocalizes with AtSUMO1/2 in the nucleus[35]. Apart from its enzymatic functions, SCE1 plays a key role in the SUMO conjugation cascade by establishing multiple interactions with the E1 activating enzyme, the target substrate, E3 ligases and SUMO through dedicated surfaces. A possible outcome of these interactions, which are mutually exclusive, is the incorporation of “directionality” to the conjugation cascade[36]. Moreover, SUMO-E2 interactions are also needed for poly-SUMO chain formation in the SUMO system [28, 37, 38].

#### E3 ligases

SUMO E3 ligases facilitate the transfer of activated SUMO from E2 to the target substrate although the requirement of E3 is not essential for in vitro SUMO conjugation to some substrates. SUMO E3 ligases do not form a thioester bond with SUMO but bind to the SUMO conjugating enzyme E2 and functions as a bridge or adaptor between E2 and the substrate to promote the transfer of SUMO to the substrate [39]. In animal systems, several E3 ligases have been identified and all of them contain SIMs [40]. The most conserved SUMO E3 ligases belong to the Siz/PIAS family. These SUMO E3 ligases contain a Siz/PIAS RING (SP-RING) domain that resembles the RING domain found in ubiquitin E3 ligases and this domain is essential for their activity and responsible for E2 recruitment[41]. The SAP domain of canonical Siz/PIAS is involved in DNA binding[42], whereas the PINIT (Pro-Ile-Asn-Ile-Thr) motif is involved in binding to SIZ1-dependent substrates. A SUMO interacting motif (SIM) present in SIZ/PIAS also contributes to the E3 ligase activity[43]. In Arabidopsis, three E3 SUMO ligases have been identified; SIZ1, MMS21 (HPY2) and PIAL1/2 (protein inhibitor of activated STAT-like 1/2). All of them belong to the SP-RING family. SIZ1 is the most well-characterized E3 ligase, and as we will see later, it is also the most frequently involved SUMO E3 ligase in SUMO conjugation dependent modulation of transcription factor functions during various biological processes. SIZ1 in plants contain an additional domain known as the PHD (plant homeodomain) domain, that is absent in animals and yeast. The PHD domain is a small protein domain that is found in nuclear proteins that interact with chromatin. Identified first as a conserved region consisting of eight regularly spaced cysteine or histidine residues in two plant homeodomain proteins (arabidopsis HAT3.1 and maize Hox1a) [44], intensive research on the molecular function of PHD domains for the past 2 decades since then has revealed that PHD domains are readers of post-translational modifications of histones. The PHD domain of two proteins BPTF and ING2 was first shown to interact with histone H3 specifically when the lysine 4 residue of H3 was trimethylated (H3K4me3) [45–48] and since then ~ 20 PHD fingers have been identified as PTM readers of di-and trimethylated H3K4 (H3K4me2/3) and also the unmethylated state of H3 (H3K4me0). Other PHD fingers interacting with histone H3 trimethylated at lysine 9 and 36 (H3K9me3 and H3K36me3) have also been reported [49–51]. The intact PHD domain of SIZ1 has been reported to be essential for the SUMOylation of a bromodomain in the GTE3 protein of Arabidopsis and, moreover, disruption of C4HC3 zinc-finger of the PHD domain of SIZ1 abolishes the ability of SIZ1 to cooperate with SP-RING domain for efficiently SUMOylating AtSCE1 in vitro [41] and is also reported to be required for the accumulation of SUMO conjugates during heat stress [52]. While these studies indicated the importance of the associated PHD domain in mediating the SUMO conjugation functions of SIZ1, recent studies have indicated that the PHD domains of SIZ1 can also perform its canonical histone tail binding functions. The PHD domain of rice SIZ1 has been reported to specifically recognize methylated Arg2 and trimethylated Lys4 of histone H3 [53] and very recently Arabidopsis SIZ1 has been shown to preferentially recognize trimethylated histone H3K4 [54]. Substitution of cysteine 162 to serine of the AtSIZ1 PHD domain abolishes its ability to bind to H3K4me3 and also has implications in the biological processes involving SIZ1, as both this C162S substituted copy of SIZ1 or a PHD domain deleted copy of SIZ1 fails to complement the *siz1* mutation phenotype characterized by growth retardation, ABA hypersensitivity and cold sensitivity. Moreover, the PHD domain of SIZ1 also interacts with the H3K4 methyltransferase, ATX1 and this interaction is proposed to suppress ATX1 methyltransferase functions. While still being subject to further validation, but these recent studies indicate that the PHD domain of SIZ1 may function as an essential sensor of the epigenetic landscape of chromatin and allow SIZ1 to modulate transcriptional pathways dependent on the status of chromatin. In contrast to SIZ1, MMS21 /HPY2 (High ploidy2) ligases only possess the SP-RING domain and a putative SIM. HPY2 is implicated in several biological processes in Arabidopsis including stem cell maintenance [55], cell cycle regulation [56, 57], gametophyte development [58], flowering [59] and drought response [60]. Double knockout *siz1mms21* mutations are embryo lethal [61] and genetic studies reveal that SIZ1 and MMS1 do not complement each other’s functions.

#### E4 ligases

The fate of the target protein that is SUMOylated is also determined by the multiplicity of the SUMO molecules attached to the target protein, which can also be polymeric in nature [62]. As discussed above, E3 ligases which enhance the transfer of SUMO proteins from E2 to target proteins can also have E4 elongase activity through which they can extend and promote the formation of SUMO chains on target proteins [63, 64]. In Arabidopsis, the PIAL1 and 2 (Protein Inhibitor of Activated STAT-Like 1/2) proteins have been identified as a novel type of E4-type SUMO ligases. These ligases belong to another group of SP-RING domain-containing ligases and promote SUMO chain formation [64]. Apart from the SP-RING domain, the study by Han et al. in 2016 resulted in the identification of a previously uncharacterized additional domain in PIAL1/2 that was termed as the IND (interacting) domain. The IND domain of PIAL1/2 was revealed to promote the dimerization of PIAL proteins and facilitate their interaction with each other and with morpheus molecule1(MOM1) [65]. MOM1 is a plant-specific CHD3-like protein initially identified as a unique component in transcriptional silencing by forward genetic screens [66]. The study by Han et al. [65], suggests that PIAL1/2 act as components of a MOM1 containing complex that mediates transcriptional silencing at heterochromatic regions. PIAL1/2 is also implicated in abiotic stress response and sulfur metabolism in plants [64].

#### SUMO proteases

In plants, four major classes of cysteine proteases are described in the MEROPS database: cysteine-, serine-, aspartate-, and metallo-proteases. The protease superfamily comprises 2% of the coding genes in plants [67]. Cysteine proteases are implicated in diverse biological processes of plants ranging from seed germination to senescence and can also facilitate plants to perceive and react to environmental stimuli as environmental cues can trigger changes in these proteases. A catalytic cysteine residue at the active site of cysteine proteases acts as a nucleophile for the formation of an acyl intermediate during proteolytic cleavage and hence the name “cysteine proteases” [68]. In all cysteine proteases, the active site cysteine residue is part of a catalytic triad or dyad and depending on whether the active site is a triad or dyad and the order of residues in the catalytic triad, cysteine proteases are subdivided into the three distinct clans: CE, CA, CP. The CE clan cysteine proteases possess a catalytic triad with residues in the order histidine, glutamine (or asparagine), and then cysteine whereas in the cysteine proteases of the CA clan, the orientation of the residues of the catalytic triad is opposite to that of the CE clan with the order of the residues being cysteine, histidine and asparagine / aspartic acid. Cysteine proteases of the CP clan on the other hand possess a catalytic dyad composed of histidine and cysteine. Till date, all identified SUMO proteases are cysteine proteases and are divided into three distinct families: ULP, DeSI, and USLP1.

#### ULPs

Ubiquitin like-specific proteases (ULPs) are members of the CE clan of cysteine proteases and belong to the ULP family. ULPs mediate SUMO maturation and deconjugation of SUMO from the target proteins through their endopeptidase and isopeptidase activities, respectively [69] and possess specificity towards both the SUMO isoform and target substrate[69–75]. ULPs are generally organized into a bipartite structure containing a C-terminal domain with a catalytic triad Gln/Asp -His- Cys (ULP/C48 domain) and a highly divergent N-terminal domain that is implicated in the regulation of ULP activity in vivo. The active site of ULPs contains a signature papain-like fold that is found in all ubiquitin-specific and UBL (ubiquitin-like)-specific cysteine proteases [71, 72]**.** The Arabidopsis genome has been predicted to encode eight ULPs and six amongst them have been characterized as SUMO proteases. ESD4 (early in short days 4) was the first characterized ULP in Arabidopsis, with the protein being identified first by Reeves et al. [73] through mutagenesis studies.ESD4 mutant plants show early flowering phenotype and reduced abundance of the floral repressor, FLC (Flowering Locus C) mRNA. Functional characterization of ESD4 as a SUMO protease was done first by Murtas et al. [74] by sequence-based homology search and in vitro assay of the peptidase activity of purified ESD4. Arabidopsis esd4*-1* mutants also have an increased abundance of SUMO conjugates and reduced levels of free SUMO. The yeast genome encodes for two ULP proteins, ULP1 and ULP2, and the yeast ULP1 (ubiquitin-like protease 1) protein was the first isolated SUMO protease through an activity-based screen of *S.cerevisiae* [75]. BLAST-based search of Arabidopsis proteins with sequence similarity to animal and yeast ULP1 catalytic domains by Kurepa et al. [25] resulted in the identification of *Arabidopsis thaliana* SUMO proteases with sequence similarity to yeast ULP1. Twelve genes were found from this search and these genes were further classified into three sub-families, with two subfamilies more related to yeast ULP1 and the third more similar to yeast ULP2. ELS1 (ESD4-like SUMO protease1) and ELS2 were identified from this study and were name ULP1a and ULP1b, respectively. The OTS1 and OTS2 ULPs were also initially identified from the study by Kurepa et al. [25], and were named ULP1d and ULP1c, respectively. The SUMO protease activity of OTS1 and OTS2 were demonstrated in vitro independently by Colby et al. [69] and Chosed et al. [70]. The study by Colby et al. [69] also demonstrated that in addition to the isopeptidase activity OTS1 and OTS2 also possess peptidase activity capable of generating the mature form of SUMO. While the search of the Arabidopsis genome for proteins similar with yeast ULP1 resulted in the identification of ESD4, ELS1, ELS2, OTS1 and OTS2 SUMO proteases; the search for proteins encoded by the Arabidopsis genome that were similar to yeast ULP2, resulted in the initial identification of SPF1 and SPF2 (SUMO protease related to fertility 1/2) by Novatchkova et al. [26]. These proteins were initially called ULP2- like-2 and ULP2-like-1, respectively. However, functional characterization of the SUMO protease activity of these proteins was done only recently by Liu et al. [76] and Kong et al. [77] and these proteins were renamed as SPF1 and SPF2 by Liu et al. [76]; while Kong et al. [77] renamed ULP2-like-2 as ASP1 (*Arabidopsis* SUMO protease1). The endopeptidase activity of SPF1 was confirmed through assessment of the cleavage of SUMO from SUMOylated FLC by wild type SPF1 [77] and this study also revealed that both SPF1 and SPF2 can process immature SUMO to the mature form. With the human SENP1 (which has a similar structural organization of bipartite N and C terminal domains like yeast ULP1) as query, FUG1 (fourth ULP gene) was identified as an expressed sequence tag (EST) in Arabidopsis [78]. FUG1 was classified to fourth ULP gene class (see below) due to its different phylogeny. Both ELS2 and FUG1 await experimental validation of their functions. Classification of the plant ULP proteins into yeast ULP1-like and ULP2-like classes had initially resulted in some ambiguity because of the disparity between the sequence conservation and the location of the ULP domain of the plant ULP proteins in comparison to yeast ULP1 and ULP2 proteins. For instance, while ESD4, ELS1/ULP1a, ULP1b, OST1/ULP1d, and OST2/ULP1c were initially classified as ULP1-like SUMO proteases, and SPF1/ASP1 and SPF2 as ULP2-like [26, 78]; ULP1d/OST1 and ULP1c/OST2 were found to be closer to yeast ScULP2 according to sequence conservation data [79], although their ULP domain is located at their C-terminus as in the ULP1-like class. Thus, an in-depth phylogenetic analysis that included Arabidopsis, poplar, grapevine and tomato genomes re-classified ULP proteins into four distinct groups in Arabidopsis, namely A, B1, B2 and C [80]. Group A contains FUG1 (At3g48480) that is yet to be characterized as a SUMO protease by experimental evaluation. ULP1d/OTS1 and ULP1c/OTS2 constitute group B1 whereas the recently characterized SPF1/ASP1 and SPF2 are classified into group B2. SPF1 and SPF2 proteins of group B2 contain the ULP domain in the middle of the protein as in the initially classified ULP2-like class. The final group, group C constitutes of ESD4, ULP1a/ELS1 and ULP1b/ELS2 and all these proteins have their ULP domain at their C-termini.

#### DeSI (DeSUMOylating isopeptidase)

The DeSI SUMO proteases, DeSI1 (deSUMOylating isopeptidase 1) and DeSI2 (deSUMOylating isopeptidase2) constitutes a novel type of SUMO proteases lacking sequence similarity to ULP enzymes and were identified first as a SUMO protease in mouse [81]. The mice DeSI proteins were identified as interactors of BZEL (BTB-ZF protein expressed in effector lymphocytes) in a yeast two-hybrid screens using as BZEL as bait. From a functional standpoint, while DeSI1 was unable to deubiquitinate ubiquitinated BZEL, it was able to deSUMOylate SUMOylated BZEL and additionally it was also capable of cleaving poly SUMO2/3 chains from targets in the mouse. However, DeSIs lack SUMO processing peptidase activity required for SUMO maturation [81, 82]. Unlike the ULP proteins, there is no homologue of DeSI in yeast. In Arabidopsis eight putative DeSI proteases were identified based on their sequence similarity to human DeSI1/2 protein and one amongst them, DeSI3a was functionally characterized [83]. The deSUMOylation activity of DeSI3a was assayed in vitro and only WT DESI3a was able to cleave isopeptide-linked SUMO in comparison to a mutated version of the protein where the catalytic cysteine was replaced by a serine. Moreover, WT DeSI3a was also shown to specifically cleave SUMO from the higher molecular weight SUMO-conjugated isoforms of the kinase domain of FLS2 (flagellin-sensitive 2) [83]. All the identified DeSI proteins of Arabidopsis belong to the CP clan [84]**.**

#### Ubiquitin-specific protease- like 1(USLP1)

USLP1 is a recently identified SUMO protease in humans that has been shown to bind to SUMO2 but not to ubiquitin and has also been shown to possess peptidase activity and some chain editing activity [85]. Currently, however, no functionally validated homologues have been identified in Arabidopsis although two Arabidopsis proteins UBP6 and UBP7 (ubiquitin-specific protease 6/7) have been identified as potential matches when the catalytic site of USLP1 was BLASTed into the Arabidopsis genome. While both UBP6 and UBP7 has been identified as ubiquitin proteases through bioinformatic analysis, and UBP6 has been shown to be an interacting partner of CAM2 (Calmodulin2) [86]. The ubiquitin protease activity of UBP6 has not been demonstrated yet, and thus the function of UBP6 and UBP7 as SUMO proteases is subject to speculation and functional characterization. If however, post rigorous validation if the functions of UBP6 and UBP7 are established as SUMO proteases, then in plants there will be representative members of all the classes of the various components of the SUMO conjugation and deconjugation machinery, implicating for the functional conservation across kingdoms of the key components involved in a dynamic post-translation modification, namely SUMOylation. For more detailed description on the different classes of SUMO deconjugases and the evolutionary relationship between them, see the excellent review by Morrell and Sadanandom [87].

## The plant SUMO cycle: outcome of the dynamics of the SUMO conjugation and deconjugation machinery

**Fig. 1:**
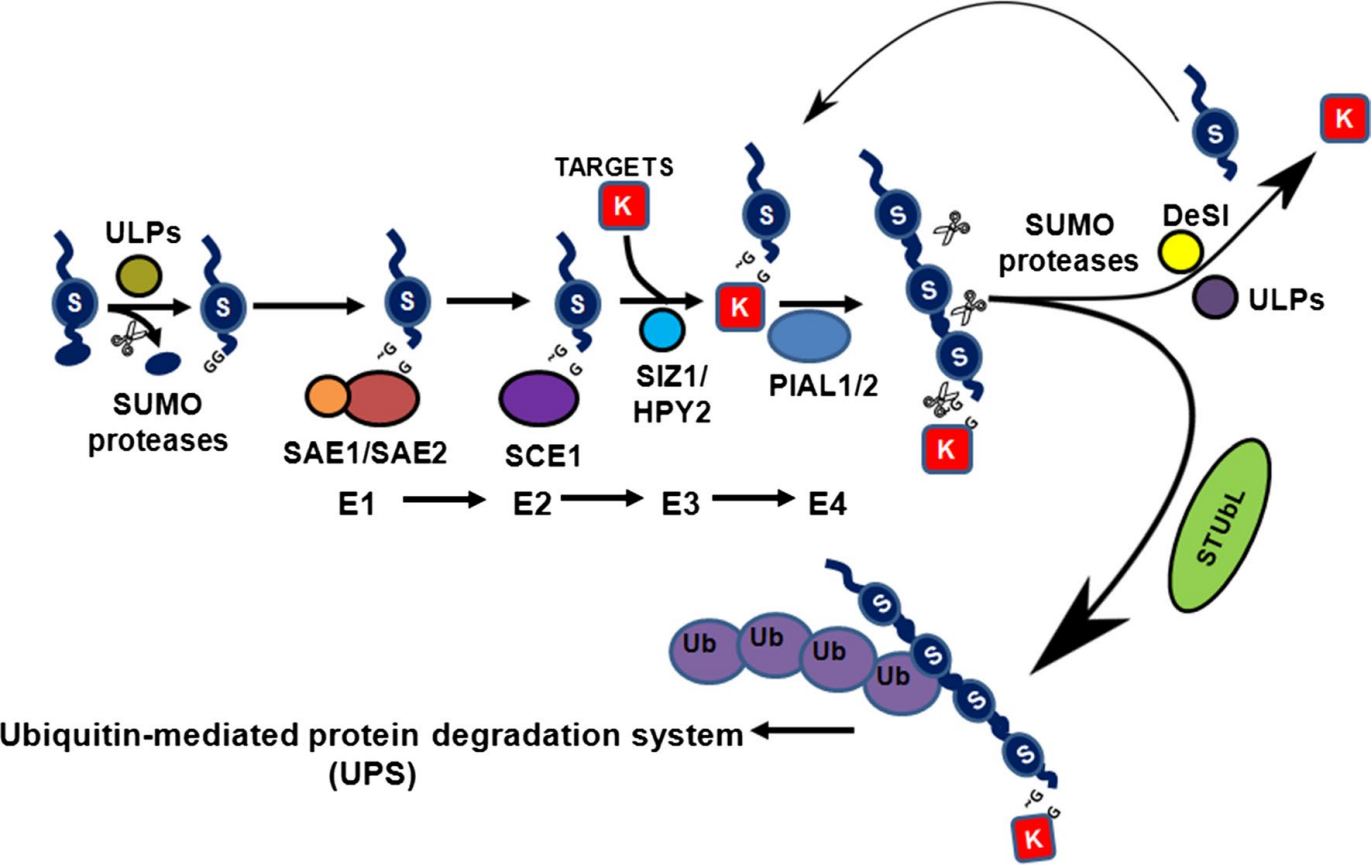
The SUMO cycle. The SUMO cycle ensues with the processing of pro-SUMO to its mature form by removal of the C-terminus of SUMO through the action of SUMO proteases. Mature SUMO (blue circle denoted as “S” in figure) is then activated in an ATP dependent step requiring the enzymatic functions of a heterodimeric complex of SAE1 and SAE2. The activated SUMO is then transferred to SCE1 (SUMO conjugating enzyme) in a conjugation step, and through the action of SUMO ligase is conjugated onto the substrate. This substrate can then be SUMOylated at more than one SUMO site or form a polySUMO chain. PolySUMOylated substrates can be polyubiquitinated through the action of specific SUMO targeted Ubiquitin Ligases (StUbLs) and targeted for degradation by the 26S proteasomal pathway. Lastly, in the deSUMOylation step, SUMO is removed from the substrate through the action of a SUMO isopeptidase to generate free SUMO. The action of the deSUMOylating isopeptidases allows the whole process to be cyclical and maintain the turnover of free SUMO

The dynamicity of SUMOylation as a post-translational modification arises at least in part from the fact that the process is cyclical. Essentially, a cascade of enzymatic steps that are biochemically similar to ubiquitination is involved in the SUMO cycle by which both the turnover of conjugated and non-conjugated free SUMO and the pool of SUMO conjugated protein is maintained during the course of the cell cycle and in response to exogenous stimuli (Fig. 1). These major steps involve activation, conjugation, ligation and de-conjugation. Activation of SUMO requires SUMO to be in its mature form. The mature form of SUMO is generated by the proteolytic cleavage of ten amino acids by SUMO peptidase, resulting in the exposure of a carboxyl-terminal diglycine motif [18]. This is followed by the activation of mature SUMO by SUMO specific E1 activating enzyme, which as discussed above is a heterodimer of SAE1 and SAE2, and this heterodimeric complex catalyzes a three-step biochemical reaction that initiates with the adenylation of SUMO and release of pyrophosphate from ATP (adenosine -5′-triphosphate) in the presence of magnesium, resulting in the formation of a high energy SUMO-acyl adenylate intermediate. In the next step, a high energy thioester bond formation takes place between the SAE2 component of E1 and the C-terminus of SUMO with the release of AMP (adenosine monophosphate) in a reaction driven by the thiol (S) group of the cysteine present at the active site of SAE2. This reaction requires a major rotation of the cysteine domain [88].The next step involves the transfer of the activated SUMO from E1 to a cysteine residue at the active site of an E2 conjugating enzyme, SCE1 (SUMO conjugating enzyme1), in a transesterification reaction resulting in the formation of SUMO-SCE1 thioester intermediate. The C-terminal tail of SAE2, although not required for in vitro SUMO activation [33], contains molecular signals that determine the subcellular localization of E1 in mammals [89], humans [90] and plants [35]. The SUMO-SCE1 thioester intermediate promotes the transfer of SUMO to a target substrate protein through a mechanism involving the nucleophilic attack at the SUMO-E2 thioester by a lysine residue of the substrate protein [91]. This lysine residue is typically part of a consensus motif for SUMOylation, *ψ*KXE/D; *ψ* denotes a large hydrophobic residue, K denotes the target lysine residue, X denotes any amino acid residue and E/D denotes glutamate or aspartate. While this step of SUMO conjugation to target substrate proteins can occur unaided, it is often mediated by SUMO E3 ligases that can interact with SCE1 and facilitate the transfer of SUMO from the SCE1-SUMO intermediate to the target protein. While the concerted action of the biochemical steps discussed above results in SUMOylation of the target proteins, the cyclical nature of the SUMO cycle is conferred upon it by the action of SUMO proteases. SUMO proteases can cleave SUMO from target substrate proteins; thereby not only making SUMOylation a reversible modification but also acting as a critical determinant for the maintenance of the pool of free SUMO. Secondly, with the cellular pools of unconjugated SUMO being very low [18], they provide a critical pool of unconjugated mature SUMO by cleaving a C-terminal peptide from newly synthesized immature SUMO through their hydrolase/peptidase activity. Finally, in the context of the SUMO cycle, the SUMO proteases maintain the equilibrium of the cycle and the status of the SUMOylome of a cell largely depends on the activity of the SUMO proteases. In contrast to the ubiquitin system, where it is believed that the specificity and the diversity of the system are conferred by a large number of E3 ligases, in the SUMO system the number of E3 ligases is few, while the number of identified SUMO proteases are relatively much higher. Since the identified SUMO proteases are specific for the target proteins the action of the SUMO proteases may not only confer specificity to the SUMO system [70, 71, 92, 93] but also determine the role of SUMOylation as a conduit for environmental influence on the signalling pathways that can control the functions of target proteins like transcription factors and chromatin modifiers required for the maintenance of phenotypic plasticity and responses to the environment in plants. Finally, SUMO modification of proteins also acts as an important tool for the regulation of protein–protein interactions. Proteins containing one or multiple copies of a motif known as SUMO interacting motif (SIM), typically consisting of several hydrophobic residues adjacent to an acidic patch of amino acid residues can bind directly with SUMO polypeptides [94] and thus can interact with SUMOylated proteins. Since such an interaction is governed by the SUMOylation status of one of the partner protein involved in the interaction, apparently the dynamics of the SUMO conjugation and deconjugation machinery dictates the interaction between the partner proteins at a post- translational level thereby adding another layer of regulation to the cell signalling pathways that may require the proteins to interact or not interact.

**Fig. 2:**
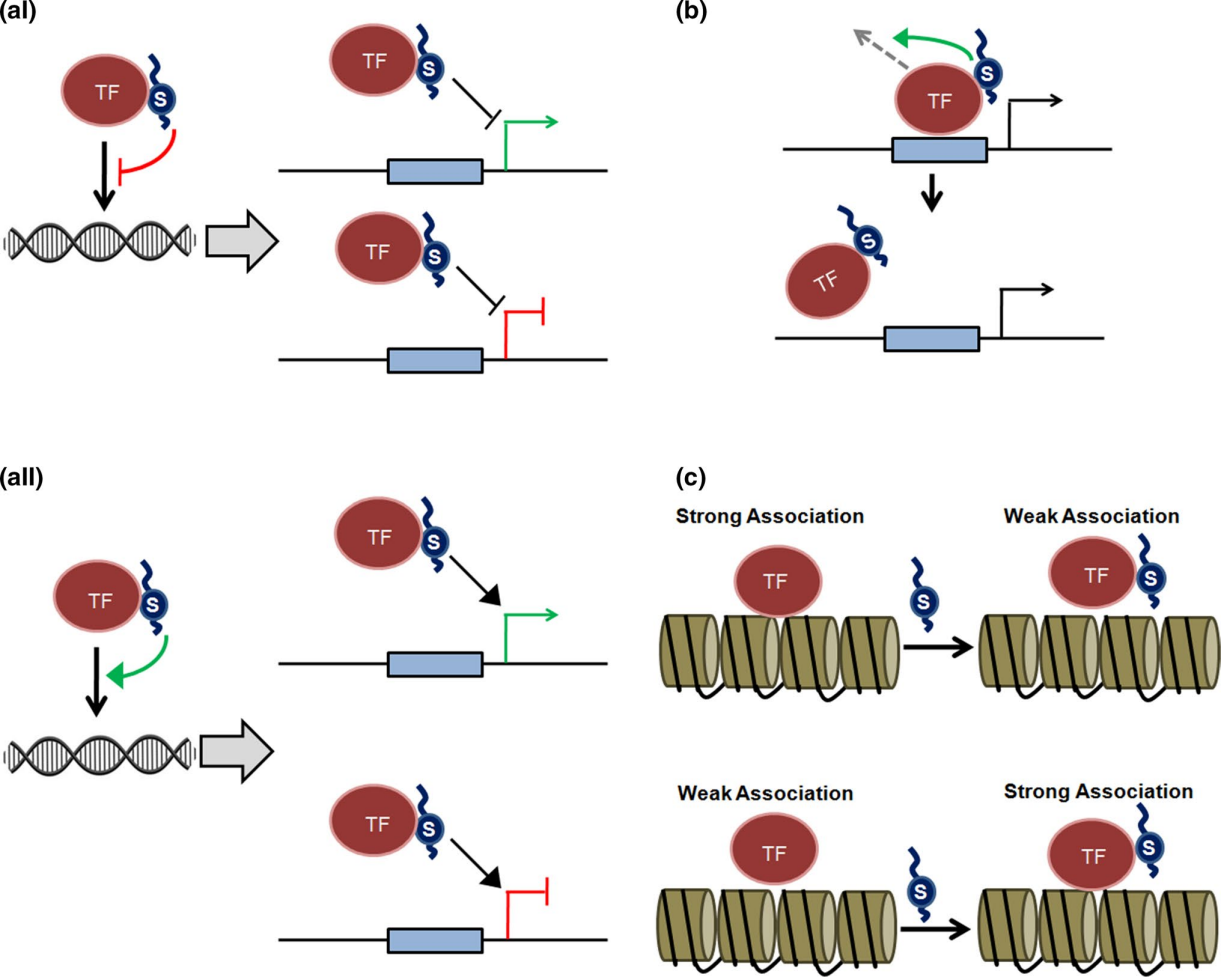
SUMO can modulate Transcription Factor functions by directly influencing the DNA binding properties of the transcription factor. **a** SUMO conjugation can either (i) inhibit or (ii) facilitate the binding of the transcription factor to its target DNA. **b** SUMO conjugation can promote the clearance of some TFs from DNA if these TFs are SUMOylated when bound to their target DNA **c** SUMO conjugation can alter the degree of association of a TF to its target site on chromatin and either alter a strong association to a weaker one (upper panel) or vice versa (lower panel) potentially by modulating the interaction of the TF with other chromatin associated co-interacting proteins. Through these mechanisms, depending on the role played by the transcription factor as an activator or repressor in the context of the cellular signalling pathway in which it is implicated, modulation of the functions of the TF by SUMO impacts the outcome of cellular signalling pathway at the level of gene expression of the downstream targets of the TF

**Fig. 3:**
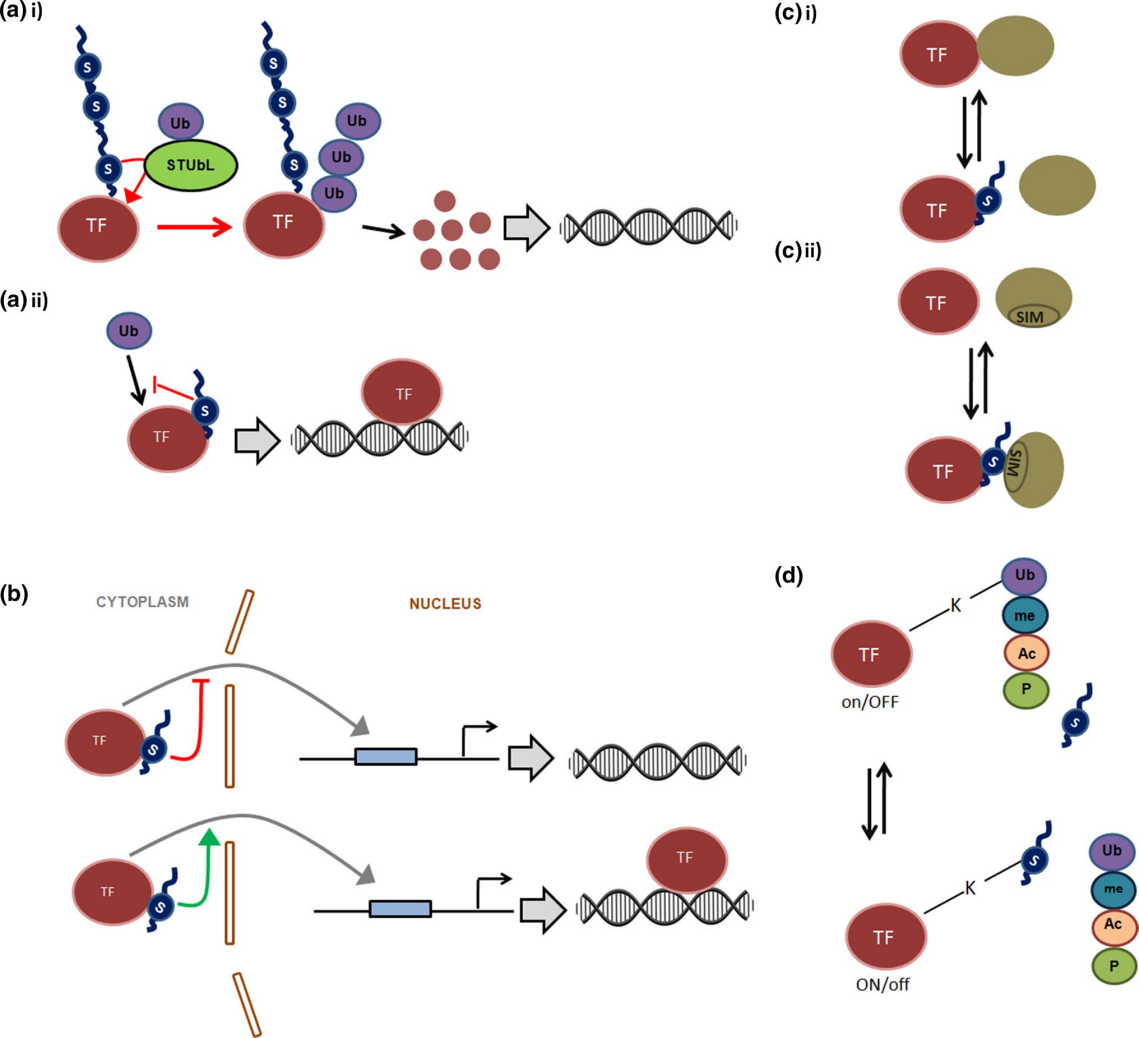
SUMO can modulate Transcription Factor functions by indirect mechanisms. **a** i) SUMO can influence the stability/ abundance of a TF either by promoting or inhibiting the degradation of the TF. For some TFs, SUMO conjugation can allow the ubiquitination of the TF through the action of StUbLs (SUMO targeted Ubiquitin Ligases) leading to consequent 26S proteasomal degradation of the TF (upper panel). **a** ii) In other cases, SUMO conjugation to TF interferes with ubiquitination of the same TF, as indicated by the red line, often due to competition for the same target lysine residues and subsequently prevents the degradation of the TF. Encircled Ub represents ubiquitin. **b** SUMOylation can regulate the subcellular localization of TFs either by inhibiting (upper panel) or promoting (lower panel) the re-localization of TFs to the nucleus. **c** SUMO can alter the capacity of TFs to interact with its partner proteins (which are often transcriptional co-regulators) by either (i) preventing the interaction or (ii) by promoting the interaction of the TF with its cognate partner through SUMO and a SIM module present in the interacting protein. In this way the dynamics of SUMO conjugation and deconjugation can influence the capacity of a TF to interact with its cognate partner and influence the regulatory output of a signalling pathway. **d** SUMO can compete with other post translational modifications like ubiquitination (ub), methylation (me), acetylation (Ac) or phosphorylation (P) for a given lysine residue of a TF. In certain scenarios (not shown in figure), SUMO can act agonistically with these post translational modifications discussed above to regulate the fate of a TF implicated in a cellular signalling pathway

In the following sections, we will assess how SUMOylation might influence the functional outcome of a signalling pathway in response to environmental cues through SUMOylation dependent modulation of transcription factor activity in plants by a detailed examination of the well-documented examples. As discussed earlier, SUMO conjugation can influence TF functions through multiple ways, either through “direct mechanisms” involving modulation of the DNA binding properties of the TF (Fig. 2) or through “indirect mechanisms” that influences the stability/abundance, subcellular localization, interaction with other proteins and the post-translational modification landscape of the TF (Fig. 3). We will thus also critically examine through these examples how SUMOylation modulates the functions of a plethora transcription factors implicated in the various cellular signalling pathways associated with different biological processes in plants both by “direct mechanisms as well as “indirect” mechanisms. We will finally assess whether these plant-specific examples reinforce the established paradigms of the mechanisms by which SUMOylation can influence transcription factor functions associated with regulation of gene expression and advocate for an evolutionarily conserved universal mechanism.

## Signalling pathways regulated by SUMO conjugation and deconjugation of transcription factors in plants

### SUMOylation of transcription factors in response to abiotic stresses

Plant’s response to abiotic stresses involves the activation of several genes whose products confer stress tolerance and on the other hand repression of several genes whose products can interfere negatively with stress tolerance. The activation and repression of these distinct classes of genes are mediated by the utilization of the activity of dedicated stress-responsive transcription factors, whose target genes form a regulon that is involved in the activation/repression of genes associated with abiotic stress responses. Amongst the described TFs encoded by the *Arabidopsis thaliana* genome, as much as 1500 are considered to be involved in stress-responsive gene expression [95]. As indicated by the transcriptome data of Arabidopsis and numerous other plants, several independent pathways respond to abiotic stress, suggesting that intricate gene regulatory networks control the transcriptional response that determines stress tolerance or susceptibility in plants[96, 97]. The increase in the abundance of SUMO conjugates in Arabidopsis plants exposed to different abiotic stresses like high salinity [98], high temperature [25, 99], freezing [100], drought [101], excess of copper [102], oxidative stress [103, 104], ethanol treatment and canavanine induced proteotoxic stress[25] indicates that there is evidently a strong correlation between SUMO conjugation and abiotic stress responses in plants. Thus, with TFs generally being a primary target for SUMOylation and with an astoundingly high number of TFs involved in abiotic stress responses, SUMO modification mediated regulation of the activity and functions of a subset of the TFs involved in abiotic stress response seems an inevitability. Some well-studied examples discussed below support this notion. SUMOylation of the transcription factor ICE1 (Inducer of *CBF/DREB1* expression) in response to cold stress provides a typical example of SUMOylation driven alteration of a specific transcriptional output in response to environmental cues facilitated by the protection of the key transcription factor involved in the process from degradation by ubiquitination and by alteration of the target specificity of the transcription factor [100]. The Dehydration Responsive Element Binding (DREB) proteins form a major class of transcription factors that regulate the expression of genes induced by cold, high salt or dehydration in plants [105–107], and can specifically bind to DNA cis-elements known as DRE/CRT elements and activate the transcription of their target stress responsive genes [108, 109]. While the expression of Arabidopsis, as well as rice DREB1/CBF (Dehydration Response Element Binding1/C- repeat Binding Factor) genes, have been shown to be induced by cold stress, *Arabidopsis* plants devoid of the SUMO E3 ligase, SIZ1 exhibit reduced expression of *DREB1/CBF*, especially *DREB1A/CBF3*. However, the expression of ICE1 is unaltered in *siz1* mutant plants indicating that SIZ1 functions downstream of *ICE1* expression [100]. ICE1 is a MYC-like bHLH upstream transcription factor of the cold signalling pathway in plants that regulates the expression of CBF genes by binding to specific sequences known as MYC recognition sequences present at the promoter region of *CBF3* and *ice1* mutation abolishes the expression of *CBF3* as well as genes downstream of *CBF3* [110]. Indeed, SIZ1 mediates the SUMOylation of ICE1 at a K393 residue and the SUMOylated form of ICE1 is specifically implicated in the activation of *DREB1/CBF3* and consequently the CBF regulon thereby leading to cold acclimatization and increased freezing tolerance. Moreover, SUMO conjugation to ICE1 also impedes the RING type E3 ubiquitin ligase, HOS1 (high expression of osmotically responsive genes1) mediated polyubiquitination of ICE1[111] and protect ICE1 from proteasomal degradation [100]. However, activation of the CBF regulon driven by SUMO conjugation of ICE1 is not characterized by a simple on–off switch as SUMO conjugation to ICE1 does not influence the transactivation activity of ICE1 as evident from GAL4 DNA binding transactivation assays[100]. Moreover, the impact of SUMOylated ICE1 on its downstream targets is not straightforward since while SUMO conjugation of ICE1 promotes *CBF3/DREB1* expression, it also represses the expression of *MYB15* [100]. MYB15 is an R2R3 MYB transcription factor that is transcriptionally induced during cold stress and acts as a negative regulator of the expression of CBF genes, including CBF3 [112]. The substantial induction of *myb15* expression during cold exposure in both *ice1* [112] and *siz1* mutants [100] also indicates that SUMOylation of ICE1 is critical for the transcriptional repression of *myb15* and it will be interesting to further assess whether SUMOylated ICE1 attenuates *myb15* expression by directly binding to the myb15 promoter as a transcriptional repressor or indirectly attenuates *myb15* expression by activating other downstream genes that can repress *myb15* expression or through interactions with other proteins that may presumably be chromatin modifiers that can alter the *myb15* chromatin landscape into a more repressive state for transcription. Similarly, since the ICE1 (K393R) substitution does not impact the transactivation activity of ICE1 in Gal4 assays but leads to the down-regulation of *CBF3/DREB1* in ICE1 (K393R) overexpressing plants, the possibility that the SUMOylation of ICE1 may influence the capacity of ICE1 to transform the chromatin landscape of *CBF3/DREB1* to a state that facilitates transcription cannot be negated. Nevertheless, repression of *myb15* and induction of *DREB1/CBF3* by SUMO conjugated ICE1 allows SUMOylation of the same transcription factor to result in both the induction as well as the repression of different sets of target genes of the transcription factor [113–116]**.** As a future perspective, identification of the specific SUMO proteases that may be implicated in the deSUMOylation of ICE1 to regulate the functions of ICE1 in the absence of cold stress will further enhance our understanding of how the regulation of the expression of cold responsive genes is intricately dependent on the dynamics of post- translational modification of transcription factors that can activate or repress cold responsive genes. Another abiotic stress related MYB transcription factor, whose activity is regulated by SUMOylation is PHR1 (phosphate starvation response1). PHR1 is a key molecular determinant required for phosphate starvation dependent responses and controls a subset of genes whose expression is activated by phosphate (Pi) limitation by binding to DNA cis elements known as P1BS (PHR1 specific binding sequence) [117–119].The lack of any changes of the transcript levels of *Arabidopsis PHR1* and the nuclear localization status of the PHR1 protein in response to any alterations in Pi status provided the initial clue that PHR1 activity might be regulated by post translational modifications [117]. Indeed, PHR1 is SUMOylated by SIZ1, in *Arabidopsis*. Lysine residues at position 261 and 372 of AtPHR1 are crucial targets for SIZ1 mediated SUMOylation of AtPHR1 and K261R and K372R substitutions impede PHR1 SUMOylation. SUMOylation by SIZ1 has been suggested to stabilize the abundance and activity of PHR1 and accelerate its binding affinity to the P1BS motif present at the regulatory region of its downstream targets such as *AtIPS1* (*INDUCED BY PHOSPHATE STARVATION 1*) and *AtRNS1* (*RIBONUCLEASE 1*) [120]. In the context of drought stress signalling in plants, the work by Srivastava et al. [121] demonstrate how SUMOylation can regulate the function of a transcription factor, OsbZIP23, that is at the fulcrum of ABA (abscisic acid) and drought stress response. This study demonstrates that the rice homolog of AtOTS1 (Overly Tolerant to Salt), OsOTS1 [122] interacts with OsbZIP23 in well-watered conditions, but drought stress induced increase in ABA levels promotes the degradation of OsOTS1. Since deSUMOylating proteases like OTS1 catalyze the cleavage of the isopeptide bond between the terminal glycine of SUMO and the lysine of the conjugated substrate, degradation of OsOTS1 results in the accumulation of SUMO conjugated OsbZIP23. The SUMO conjugated form of OsbZIP23 is then implicated in facilitating the expression of genes implicated in the promotion of drought stress tolerance and productivity in rice. The role of the *OTS1/OTS2* (*ULP1C/ULP1D*) SUMO protease genes in negatively influencing drought stress resistance has been previously demonstrated in *Arabidopsis*, where *ulp1culp1d* double mutants exhibit enhanced drought resistance in comparison to wild type plants [79] and as well as in rice, where over-expression of *OsOTS1* results in increased drought sensitivity while *OsOTS1* depleted transgenic lines exhibit drought tolerance in pot grown plants [121]. The study by Srivastava et al. [121], clearly illustrates that the turnover of SUMO conjugated form of a transcription factor can also be driven by regulation of the SUMO deconjugation machinery rather than the SUMO conjugation machinery and highlights SUMOylation of transcription factor implicated in drought stress response as an important process that explains the negative correlation between SUMO protease activity and drought tolerance in plants. Thus, this study shows how the dynamics of the SUMO conjugation/deconjugation machinery act as a critical regulator for a cellular signalling pathway implicated in the response to environmental cues, by determining the functional status of the components of the signalling pathway through SUMO conjugation or deconjugation. Apart from OsbZIP23, multiple other transcription factors related to drought and water deficit stress have been identified to be SUMOylated by proteomic studies in *Arabidopsis* [103, 104, 123]. These transcription factors include the AP2/ERF transcription factors DREB2A and ERF107; the ABA response element binding protein, ABF3 [123]; a member of the plant WRKY transcription factor family, WRKY33 [103, 123] and the homeodomain leucine zipper class I (HD-ZipI) protein, ATHB6 [103, 104, 123]. As a perspective for the future, it will be interesting to further assess how SUMOylation of these abiotic stimuli and ‘water deprivation response’ related transcription factors influence their ability to activate or repress a specific subset of target genes. Regulation of salt stress response by SUMOylation of a transcription factor has also been reported recently[124]. In response to salt stress, the R2R3 transcription factor MYB30 is SUMOylated by SIZ1 and this SUMO conjugation at lysine 283 of MYB30 is critical as indicated from the fact that K283R substitution of MYB30 results in the ablation of the ability of MYB30 to bind at the promoter of its downstream target *AOX1a* ( *alternative oxidase1a*) and up-regulate its expression. Moreover, MYB30 K283R mutant fails to rescue the salt sensitive phenotype of *myb30-2* mutant, again indicating the indispensability of SUMO conjugation for MYB30 activation of *AOX1a* expression, which in turn facilitates the maintenance of cellular redox homeostasis via AOX1a and confers salt tolerance.

### SUMOylation of the transcription factors in Brassinosteroid signalling

The plant-specific steroid hormone, brassinosteroid (BR) is implicated in the regulatory control of a variety of physiological processes ranging from seed development to flowering and senescence [125–127]. Recently, SUMOylation of BZR1 (brassinazole resistant 1), a key transcription factor of BR signalling was shown to be a critical event that allows for the modulation of growth during stress in a BR dependent manner [128]. The BR signalling pathway involves the binding of BR to the membrane receptor kinase, BRI1 (brassinosteroid insensitive1), resulting in the perception of BR by plants and triggers a signal transduction cascade that initiates with the recruitment of the co-receptor kinase BAK1 (BRI associated Kinase1) to BRI1 [129–134]. After the formation of the BAK1-BRI1complex, a cascade of phosphorylation and dephosphorylation events ensues with BRI1 mediated phosphorylation of BSK1 (BRI1 substrate kinase1) and CDG1 (constitutive differential growth 1), which in turn bind and activate the nuclear localized serine-threonine phosphatase BSU1 (BRI1 suppressor 1) by phosphorylating BSU1 [135–137]. Phosphorylated BSU1 can then inactivate BIN2 (BR insensitive 2), a GSK3 kinase, by dephosphorylating a conserved phospho-tyrosine residue (pTyr200) of BIN2 [136] and inactivated BIN2 is targeted for degradation by the proteasome [138]. Inactivation of BIN2 post BR perception is a key point of regulation in BR signalling as BIN2 in its activated state phosphorylate BZR1 and its homolog BES1(bri1-EMS suppressor 1) and cause their cytoplasmic retention and target them for proteasomal degradation [139–142]. SUMO conjugation to BZR1 impedes BZR1-BIN2 interaction resulting in the translocation of BZR1 to the nucleus, where BZR1 can promote the expression of BR responsive genes. The translocation of BZR1 to the nucleus is further facilitated by the inhibition of BIN2 by upstream BR signalling components in the presence of epi- brassinolide (BL). On the other hand, increased accumulation of the SUMO protease ULP1a during salt stress causes rapid deconjugation of SUMO from BZR1 and results in the cytoplasmic retention and subsequent degradation of BZR1 due to enhanced interaction with BIN2 [128]. In this scenario, the dynamics of BZR1 SUMOylation and deSUMOylation seems to act as a molecular switch that coordinates BR signalling and salt stress response and allows the regulation of BZR1 function to be coordinated by two different pathways involving two distinct post-translational modifications, namely phosphorylation/dephosphorylation and SUMOylation. Another example of cross talk between SUMOylation and phosphorylation implicated in the localization of a TF to nuclear bodies is also evident from the BR signalling pathway. However, in this case, the cross talk between SUMOylation and phosphorylation not only influences the localization of the concerned TF but is also directly involved in regulating the transcriptional activity and in vivo DNA binding capacity of the TF. The basic HLH transcription factor CESTA (CES) is implicated in BR responses and can bind directly to G-box motifs *in planta* [143].SUMOylation of CES at K72 residue promotes the localization of the protein to nuclear bodies and promoting SUMOylation of CES induces constitutive nuclear compartmentalization. Phosphorylation of serine 75 and 77 of the identified novel extended SUMOylation motif antagonizes SUMOylation at K72 residue of CES. Impairment of SUMOylation either by K72R substitution or phosphorylation at S75 and S77 decreases both the transcriptional activity evident from transactivation assays of CES as well as in vivo DNA binding capacity of CES evident from the decreased occupancy of CES at the promoter of its target BR biosynthetic gene, *CPD* indicated by chromatin immunoprecipitation assays [144].

### SUMOylation of transcription factors in abscisic acid (ABA) signalling

The ABA- responsive bZIP transcription factor, ABI5 (abscisic acid insensitive 5) plays a central role in abscisic acid signalling by regulating the expression of downstream genes that harbour the ABSCISIC ACID RESPONSE ELEMENT (ABRE) motif at their promoter regions [145]. A complex and dynamic interplay of phosphorylation, ubiquitination and SUMOylation is involved in the regulation of ABI5 functions. Phosphorylation of ABI5 by SNF1 related protein kinases (SNRK2.2, SNRK2.3 and SNRK2.6) promotes the transcriptional activity of ABI5 post perception of ABA by the ABA receptor [146]. However, in the absence of ABA, ABI5 is targeted for degradation by the 26S proteasome driven by the ubiquitination of ABI5 in the cytoplasm by the E3 ubiquitin ligase KEEP ON GOING (KEG) [147, 148] and in the nucleus by Cul4-based E3 ubiquitin ligases [149, 150], resulting in the consequent maintenance of ABI5 at low levels. SUMOylation of ABI5 at lysine 391 by SIZ1, on the other hand, protects ABI5 from degradation [151] through a mechanism that cannot be simply explained as a consequence of the competition between the cellular ubiquitination and SUMOylation machinery for the same site on the target protein, as these PTMs occur at different sites of ABI5. However, curiously, SUMOylation of ABI5 results in negative regulation of ABA signalling as evidenced from the fact that loss-of-function *siz1* mutant plants display hypersensitivity to ABA for inhibition of seedling primary root growth and seed germination and hyper induction of ABI5 downstream target genes such as *RD29A, RD29B, AtEm6, RAB18, ADH1*. Furthermore, expression of a mutant K391R transgene of ABI5 that cannot be SUMOylated in *abi5* loss of function mutants also resulted in increased ABA hypersensitivity and ABI5 downstream target gene expression [151]. Thus, SUMOylation driven negative regulation of the intrinsic transcriptional activity of ABI5 by a yet to be deciphered mechanism and the simultaneous protection of ABI5 from degradation indicates that SUMOylation influences the abundance of ABI5 and maintains it in an inactive state. As proposed by Miura and Hasegawa [152], this may allow SUMOylation to act as a conduit for the regulation of ABA-dependent responses as the storage of ABI5 in its inactive form by SUMOylation allows the process to be reversed by the deSUMOylation machinery, thereby converting ABI5 into its active form when necessary. Therefore, SUMOylation dependent maintenance of the threshold levels of ABI5 in its inactive form, and the consequent activation of the protein by deSUMOylation potentially in response to ABA, also provides for a mechanism that can allow the bypass of the need of active transcription of *ABI5* gene in response to ABA. With a relatively large number of plant SUMO proteases displaying target protein specificity, future identification of specific SUMO proteases implicated in the regulation of ABI5 functions will provide the final missing piece to the puzzle of regulation of ABI5 functions by SUMOylation/deSUMOylation. SUMOylation of the transcription factor MYB30 by SIZ1 also has implications on ABA signalling [153] apart from salt stress response discussed previously. MYB30 has been shown to function in a pathway parallel to the ABI5 pathway to coordinately regulate ABA response and the downstream target genes of MYB30 are different from that of ABI5 [153]**.** Like ABI5, SUMOylation of MYB30 at lysine 283 by SIZ1 prevents its degradation and increases the stability of the protein. However, in contrast to ABI5, SUMOylation of MYB30 positively impacts its transcriptional activity *in planta* as evidenced from the fact that in contrast to *MYB30,* expression of *MYB30K283R* does not rescue the expression of downstream target genes *TAT3*, *LOX3*, *BGL2*, *bHLH*, C*OR15b* and *COR413* and does not fully restore ABA sensitivity in *myb30* plants. While previously, the importance of regulation of the transcriptional activity of MYB30 by the degradation of the protein by RING E3 ligase MIEL1 (MYB30-Interacting E3 Ligase1) mediated ubiquitination has been demonstrated in hypersensitive cell death response and defense in plants [154], in the context of ABA signalling, degradation of MYB30 by the recently identified RING-type ubiquitin E3 ligase RHA2b has been shown to be implicated in the regulation of MYB30 functions [155]. RHA2b positively regulates ABA signalling by interacting with MYB30 and ubiquitinating it thereby influencing the stability and abundance of the protein through the 26S proteasome pathway. Lysine 283 and lysine 165 are critical sites for ubiquitination of MYB30 in ABA-induced degradation. As discussed previously, the lysine 283 residue is also the target site for SIZ1 mediated SUMOylation of MYB30. Thus, regulation of the functions of MYB30 in ABA associated responses indicates how SUMOylation and ubiquitination can act antagonistically by competing for the same residue of the target protein for modification. Therefore, co-regulation of MYB30 stability during ABA response by ubiquitination and SUMOylation allows these two PTMs to fine-tune MYB30 functions according to the environmental signal.

### SUMOylation of transcription factors in Auxin signalling

The plant hormone, auxin, regulates lateral root development [156] apart from fine-tuning of many developmental processes in plants [157–160]. Spatiotemporal changes in auxin levels trigger rapid and precise reprogramming of downstream target genes through the action of early auxin response genes such as the Auxin/Indole-3-Acetic Acid (Aux/IAA) family, the auxin response factor (ARF) family, glutathione-*S*-transferase (GH2/4-like), aminocyclopropane-1-carboxylic acid synthase (ACS), the auxin-responsive Gretchen Hagen3 (GH3) family and small auxin upregulated RNA (SAUR) [159, 161, 162]. SUMOylation of the ARF family transcription factor, ARF7, which is a core component of the auxin response machinery, is implicated in a physiological response in plants termed “hydropatterning” [163]. “Hydropatterning” is an adaptive root branching response involving the formation of lateral roots when roots are in direct contact with moisture [164, 165]. ARF7 is implicated in the regulation of lateral root (LR) initiation [156, 166–168] and controls the auxin-dependent expression of LR regulatory genes like *LBD16*. Increased occupancy of ARF7-^4K/4R^-GFP (a GFP tagged non SUMOylatable version of the protein where the target lysine residues for SUMO conjugation are mutated to arginine) at the promoters of its downstream targets *LBD16* and *LBD29* in comparison to wild type ARF7-GFP indicates that SUMOylation of ARF7 negatively regulates its auxin-induced DNA binding activity [163]. Indeed, higher DNA binding of ARF7-^4K/4R^-GFP at the promoters of *LBD16* and *LBD29* in comparison to ARF7-GFP also indicates that SUMOylation positively influences ARF7 promoter clearance. The negative impact of SUMOylation on the transcriptional activity of ARF7 is attributed to the function of SUMO as an essential mediator for the interaction of ARF7 with the Aux/IAA (indole -3-acetic acid) repressor protein, IAA3. The SUMO interacting motif (SIM) of IAA3 is critical for the interaction of IAA3 with SUMOylated ARF7 and this interaction results in the creation of a transcriptional repressor complex that can block the expression of auxin-responsive genes on the airside of roots, where there is non-availability of water. Conversely, maintenance of ARF7 in its non –SUMOylated form on the water contact side putatively by the action of the SUMO protease OTS1, allows induction of ARF7 target genes like *LBD16* to trigger lateral root organ initiation. The regulation of ARF7 transcriptional activity by SUMOylation illustrates how SUMO conjugation can influence the function of a TF by modulation of the interactions of the TF with a co- repressor protein resulting in a change in the status of the TF from an activator to a component of a repressor complex. SUMOylation can also have the opposite effect on the status of a TF by promoting the association of the TF with transcription co-activators as evident from the SUMOylation dependent regulation of the function of the master regulator of Salicylic acid (SA) responsive genes, NPR1(NON EXPRESSOR OF PATHOGENESIS RELATED GENES1), which is discussed in detail in the following section.

### SUMOylation of transcription factors in Salicylic acid (SA) responses and plant immunity

The low molecular weight phenolic compound, Salicylic acid (SA) is a critical signalling molecule in plant defense response, required for both local and systemic immunity in plants, a phenomenon known as systemic acquired resistance (SAR), and is therefore widely regarded as the key plant immunity hormone. The NPR1 (NON EXPRESSOR OF PATHOGENESIS RELATED GENES1) protein acts as a master regulator of SA signalling as it connects the initial step of the SA signalling pathway i.e. the perception of SA, to the final stages of the pathway involving activation of the expression of pathogenesis-related (PR) genes. The expression of *PR* genes is essential for SAR and NPR1 plays a critical role in this process as a positive regulator of *PR* gene expression and thereby SA response. Indeed, *Arabidopsis* mutants deficient in NPR1 exhibit reduced expression of *PR* gene and increased susceptibility to pathogens [169, 170] and on the other hand, constitutive expression of *NPR1* in wild type *Arabidopsis thaliana* ensures a quick response to salicylic acid. In the absence of SAR, NPR1exists as an oligomer formed through intermolecular disulfide linkages and is sequestered from transport to the nucleus. SA-regulated immunity involves SAR induction triggered monomerization of NPR1 and localization of NPR1 to the nucleus [171], where it indirectly activates *PR* gene expression by interacting with a class of bZIP transcription factors called TGAs (TGACG motif binding protein family) [172–175]. Within the complex landscape of post-translational modifications of NPR1, SUMOylation plays a critical role in the regulation of NPR1 functions by increasing the association of NPR1 with TGA transcription factors[31]. Unmodified NPR1 associates with the WRKY70 transcriptional repressor and mediates repression of SA responsive genes such as the well characterized gene *PR1* by localizing to the WRKY DNA binding motif, W box present at the promoter of *PR1*. Conjugation of SUMO3 to NPR1 upon SA induction, on the other hand, promotes the dissociation of NPR1 from both WRKY and the W-box motif as evident from ChIP assays where conversion of NPR1 to a non SUMOylatable form results in its constitutive localization to the W box motif of *PR1* promoter. Parallelly, SA induced SUMOylation of NPR1 dramatically increases the association of NPR1 with the *as-1* element present at the *PR1* promoter through SUMO3 mediated interactions of NPR1 with TGA3 [31]. In the absence of SA, nuclear NPR1 is thought to be maintained at low levels by proteasomal degradation of NPR1 triggered by CRL3^NPR4^ mediated ubiquitination of NPR1 and this prevents the activation of immune response genes [176–178]. An increase in SA levels due to immune activation results in the phosphorylation of Ser11/15 residues present within the N-terminal IκB-like phosphodegron motif of NPR1 and this, in turn, promotes the ubiquitination and turnover by the alternate E3 ligase CRL3^NPR3^. Although paradoxical, this degradation of NPR1 mediated by the alternate E3 ligase CRL3^NPR3^ is necessary for the full induction of the target genes of NPR1 [177, 178]. Evidently, the phosphorylation of NPR1 at Ser11/15 has a positive effect on NPR1 SUMOylation as phospho- mimic npr1 ^S11D/S15D^ exhibits enhanced interaction with SUMO3 that leads to further SUMOylation. On the other hand, SUMO3 also promotes phosphorylation of Ser11/15 of NPR1, and thus by these mechanisms SUMOylation amplifies the transcriptional output from the SA signal by coordinately inducing the function of NPR1 as a transcriptional co-activator and by maintaining the turnover of NPR1that is in turn required for the full activation of its downstream targets. SIZ1 mediated SUMOylation of the transcriptional co-repressor TOPLESS-RELATED1 (TPR1) is also implicated in plant immune response [179]. TPR1 functions as a transcriptional co-repressor and associates with the histone deacetylase HDA19 (HISTONE DEACETYLASE 19) and positively regulates plant immunity by mediating the repression of genes encoding negative regulators of plant immune response such as *DND1* (*DEFENSE NO DEATH1*) and *DND2*. In this regard, SUMOylation of TPR1 at lysine 282 and lysine 721 residues influences TPR1 mediated plant immune response by inhibiting its co-repressor activity putatively by repressing its interaction with HDA19. Since histone deacetylases remove acetyl groups from ε-N-acetyl-lysine residues from histones thereby allowing histones to wrap DNA more tightly, inhibition of the interaction of HDA19 and TPR1 may confer a more “open” chromatin state at the regulatory regions of the target genes of TPR1 thereby facilitating the transcription of these genes such as *DND1* and *DND2*. Thus evidently, SUMOylation mediated modulation of TPR1 functions indicate that in plant systems also SUMOylation can alter the capacity of the TF to interact with chromatin dependent transcriptional regulators like histone deacetylases (HDAC).

### SUMOylation of DELLA protein regulates plant growth independently of GA (gibberellins)

The plant hormone, gibberellin, mediate regulation of many growth and developmental aspects throughout the life cycle of plants that includes promotion of cell division and elongation, elongation of stem and roots, bolting, control of seed dormancy and germination, and promotion of flower and fruit development and responses to biotic and abiotic stresses [180–183]. The DELLA (aspartic acid–glutamic acid–leucine–leucine– alanine) proteins are a subfamily of the plant-specific GRAS (GIBBERELIC ACID INSENSITIVE REPRESSOR OF *ga1-3* SCARECROW) family of transcriptional regulators that mediate gibberellin (GA) signalling and function as key repressors of molecular pathways that are governed by GA [184–187]. Recent pieces of evidence indicate that as an adaptive strategy for survival during adverse conditions, plants restrain growth via accumulation of DELLA [188–192] and studies over the past decade have indicated that the ability of DELLAs to function as transcriptional regulators is largely due to their interactions with a diverse battery of transcription factors [193]. For instance, the DELLA protein from Arabidopsis, GAI (gibberellic acid insensitive) has been recently described to interact with at least 57 different transcription factors [194]. A direct consequence of this interaction of DELLA proteins with multiple transcription factors is the modulation of the activity of the transcription factors bound by DELLAs as seen for the transcription factors: PIFs, BZR1, EIN3( ETHYLENE INSENSITIVE3) and RELATED TO APETALA2.3 (RAP2.3), where DELLAs binds directly to the DNA binding domains of these transcription factors and block their activity [195–202]. SUMOylation of the DELLA proteins, primarily RGA (REPRESSOR OF *ga1-3*) has been shown to allow plants to control growth independently of gibberellin [203]. Amongst the DELLA proteins, RGA has a broader domain of expression and predominant effect in the control of GA growth and development [187, 204] and null mutations of RGA partially alleviates several GA deficiency defects indicating the repressive role of RGA in GA-mediated processes. As illustrated in the study by [203], SUMOylation of a pool of DELLA proteins increases the stability and abundance of non-SUMOylated DELLA proteins and consequently the total pool of DELLA proteins through a mechanism that interferes with the functions of the GA receptor GID1 and the targeted degradation of the DELLA proteins by ubiquitination associated with the functioning of GID1. GID1 contains a SIM site at its N-terminus that coincides with the interface that forms the DELLA- binding site. In the presence of GA, the GID1-GA complex binds DELLA and targets it for ubiquitin-mediated proteasomal degradation in the presence of another protein known as SLEEPY1.In the absence of GA, accumulation of a pool of SUMOylated DELLA results in the binding of SUMOylated DELLA to GID1 through SUMO-SIM interaction thereby occluding the binding interface of GID1 for non- SUMOylated DELLA. This results in the sequestration of GID1 through a mechanism that is not strictly dependent on GA. As demonstrated in this study, the SUMO proteases OTS1 and OTS2 play key roles in this signalling pathway by mediating the deSUMOylation of DELLA proteins that result in the decrease in the abundance of the DELLA proteins. Indeed, SUMO protease activity of OTS1 and OTS2 targeted towards DELLA may partially explain the targeted degradation of these SUMO proteases for the promotion of beneficial growth restraint during multiple stresses such as salinity [98] and drought [121] stress. In relation to the paradigms of SUMOylation dependent regulation of TF functions, this study presents a unique example where SUMOylation of a fraction from the pool of a particular transcriptional regulator positively influences the steady-state abundance of the remaining non-SUMOylated counterpart of the protein.

### SUMOylation of transcription factors/co-regulators during flowering

In higher plants, flowering involves the transition of a vegetative meristem producing leaves and stems to a floral meristem producing flowers, that requires the coordinated and sequential functions of a wide array of transcription factors and is regulated through a complex network of both genetic and epigenetic pathways. The regulation of flowering time in Arabidopsis can be controlled by four major genetic pathways. The long-day and the vernalization pathways mediate floral transition in response to environmental cues, whereas the other two pathways namely the autonomous pathway and the GA pathway function independently of environmental cues. The autonomous pathway promotes flowering under all conditions and GA pathway is required for flowering during non-inductive short-day conditions. Irrespective of the pathway employed, the final output of these genetic pathways converge in the induction of a key set of genes known as floral meristem identity genes, which promotes the specification of floral meristems on the flanks of the shoot apical meristem [205–212]. Regulation of the expression of the *FLC* (Flowering locus C) gene, which encodes a MADS box transcription factor [213] serves as a key nodal point in the regulatory network controlling flowering. Since *FLC* regulates almost all the major genetic pathways implicated in floral transition by repression of the key players of the different pathways that are involved in the activation of the floral meristem identity genes, many signals that control flowering converge in the regulation of *FLC* expression. One of the key proteins that is involved in the regulation of *FLC* expression is the autonomous pathway protein, FLOWERING LOCUS D (FLD). FLD is the plant ortholog of human KIAA0601/LYSINE-SPECIFIC HISTONE DEMETHYLASE1 (LSD1) and it represses FLC expression by facilitating deacetylation of histone H4 in FLC chromatin [214, 215]. Evaluation of the expression and histone H4 acetylation status at *FLC* chromatin in *fld*-6 protoplasts transiently expressing either *HA:FLD* or *HA: FLDK3R* (constitutively non SUMOylatable) indicated that the levels of *FLC* mRNA and the H4 acetylation levels at *FLC* chromatin were significantly reduced in protoplasts expressing the non SUMOylatable version of FLD (*HA: FLDK3R*) as compared to protoplasts transiently expressing *HA:FLD* [216]. The results from this study indicate that SIZ1 mediated SUMOylation of FLD disrupts its functions in a manner that is reminiscent of the mutations that disturb FLD functions resulting in *FLC* transcript accumulation and H4 hyperacetylation at the first intron of *FLC* [214]. Thus, SUMOylation status of FLD acts as a critical determinant of the inactive and active state of the protein and the dynamics of the SUMO conjugation and deconjugation machinery may be a key regulatory step in the control of flowering through the autonomous pathway.

Table 1 summarises the various mechanisms by which SUMOylation modulates the functions of the transcription factors discussed in this review. The biological processes impacted by modulation of the TF functions by SUMO conjugation/deconjugation is also indicated in the table.

**Table1:** Summary of the mechanisms of SUMO mediated modulation of TF functions in various cellular signalling pathways in plants

Transcription factor/co-regulator	Cellular signalling pathway	Impact of SUMOylation on TF mode of action						Impact on TF target gene expression	Reference
		A	B	C	D	E	F		
AtICE1	Cold stress response			?	+		** + **	Activation as well as repression depending on target	[85]
AtPHR1	Phosphate starvation response	** + **			** + **			Potentially activation	[105]
OsbZIP23	Drought stress response				** + **			Activation	[106]
AtMYB30	Salt stress response	** + **						Activation	[109]
AtBZR1	Brassinosteroid signalling				** + **		** + **	Activation	[113]
AtCESTA	Brassinosteroid signalling	** + **		** + **			** + **	Activation	[129]
AtABI5	Abscisic acid signalling				** + **		** + **	Repression	[136]
AtMYB30	Abscisic acid signalling				** + **		** + **	Activation	[138, 140]
AtARF7	Auxin Signalling	** + **	** + **			** + **		Repression	[148]
AtNPR1	Salicylic acid signalling/plant immunity				** + **	** + **	** + **	Activation post association with TGA	[19]
AtTPR1	Salicylic acid signalling/plant immunity					** + **		Activation	[164]
DELLA	GA signaling				** + **				[191]
AtCCA1	Circadian Clock	** + **							[205]

key: a: influencing the DNA binding activity, b: promotion of clearance of the transcription factor from chromatin, c: alteration of the association of the transcription factor to its target binding sites on chromatin, d: regulating the abundance /localization and or stability of the transcription factor e: altering the capacity of the transcription factor to interact with transcriptional co-regulators, f: interplay with other post translational modifications that can influence transcription factor activity

## Concluding remarks and future perspectives

Increasing pieces of evidence indicate that the indispensability of SUMOylation as a post-translational modification implicated in growth, development, maintenance of phenotypic plasticity and dynamic responses to changes in the environment in plants, is largely due to SUMOylation of TFs that are essential for these processes. The list of biological processes that are influenced by SUMOylation/ deSUMOylation of transcription factors that are implicated in the molecular signalling pathways regulating the concerned biological processes in plants are ever-growing. For instance, apart from the biological signalling pathways discussed above, recent studies have indicated that SUMOylation affects the circadian clock and secondary cell wall formation in Arabidopsis. SUMOylation was shown to suppress the DNA binding activity of the morning phased central plant clock transcription factor CIRCADIAN CLOCK ASSOCIATED (CCA1) [217] while SIZ1 mediated SUMOylation of the transcriptional regulator LBD30 was shown to affect activation of the *SND1*/*NST1*-mediated transcriptional networks for SCW formation in fiber cells [218]. Large scale proteomic studies and yeast 2 hybrid-based approaches have significantly expanded the repertoire of the targets of the SUMO system in plants indicating SUMOylation to be implicated in chromatin modification/ remodelling, transcriptional activation/ repression, epigenetic reprogramming and RNA metabolism (see the excellent review by Augustine et al. [219] and references within). During the past two decades, intense research on the effect of SUMOylation on TF functions in the context of various molecular signalling pathways in plants have reinforced that SUMO modifications essentially impacts plant TFs in the same way as their animal counterparts. Consequently, considering both the differences and the similarities between the molecular players involved in SUMO conjugation and deconjugation machinery of plants and animal systems, SUMO modification mediated regulation of TF functions in plants indeed present the scenario of “different players, same rules”, thereby advocating for an astoundingly conserved post-translation modification that can influence transcription factor functions across kingdoms. The major challenge that lies in front of us now, post these two decades of research on SUMOylation, is the assessment of “what” determines the final consequence of TF SUMOylation i.e. whether SUMOylation of transcription factors will lead to the activation or repression of its targets and can we in the near future be able to predict the consequential fate of a TF due to SUMOylation? Early studies on the role of SUMOylation in the regulation of transcription in yeast and animal systems resulted in a general association of SUMOylation with inhibition of transcription although recent genome-wide studies in yeast and animal systems have nuanced this view (see review by [220] for further details). With our increasing understanding of the impact of SUMOylation of TFs on downstream target gene expression in plants, it is becoming evident that SUMO conjugation mediated modulation of transcription factor functions may impact target gene expression in either way i.e. repression or activation. Evidently, a subset of transcription factors may be activated by SUMOylation, while another subset may be de-activated. Depending on whether a transcription factor acts as an activator or a repressor in a particular signalling pathway in which it is implicated, the fate of the expression of downstream target genes of the transcription factor in the signalling pathway can be determined by how SUMOylation impacts the transcription factor functions. Another intriguing facet of SUMOylation is the multiplicity of the molecular mechanisms by which it can influence TF functions, and this allows a single post-translational modification to differentially influence the fate of a diverse array of cellular signalling pathways. This aspect of SUMOylation provides a particular strategic benefit to plants, as it allows them with a scope to intricately regulate the dynamics of multiple signalling pathways that are involved in response to a particular environmental cue through a single post-translational modification. In this way, SUMO acts as a potent conduit for the environment to influence multiple cellular signalling pathways in plants through a simple switch of SUMO conjugation/ deconjugation of target proteins which are often transcription factors. However, while the consequence of SUMOylation of a TF may vary concerning whether the target genes of the TF are activated or repressed, it remains enigmatic how through a set of conserved mechanisms, SUMOylation can differentially affect the functions of a plethora of transcription factors and in the near future research on SUMO functions may be aggravated towards the understanding of this phenomenon.
